# Antiperistaltic Transverse Coloplasty: A Salvage Procedure in Extensive Bowel and Colorectal Resections to Avoid Intestinal Failure

**DOI:** 10.1245/s10434-023-14165-0

**Published:** 2023-08-26

**Authors:** Juan José Segura-Sampedro, Rafael Morales-Soriano, José Carlos Rodríguez-Pino, Cristina Pineño Flores, Andrea Craus-Miguel

**Affiliations:** 1 General and Digestive Surgery Department, University Hospital Son Espases, School of Medicine, University of the Balearic Islands, Health Research Institute of the Balearic Islands, Palma de Mallorca, Spain; 2 General and Digestive Surgery Department, University Hospital Son Espases, Health Research Institute of the Balearic Islands, Palma de Mallorca, Spain; 3 General and Digestive Surgery Department, University Hospital Son Espases, Palma de Mallorca, Spain

**Keywords:** Antiperistaltic coloplasty, Short bowel syndrome, Quality of life, Colorectal anastomosis, Cytoreductive surgery, Colorectal techniques

## Abstract

**Introduction:**

After extensive small and colon resections, quality of life can be affected. We propose the antiperistaltic transverse coloplasty as a solution that allows for preservation of the transverse colon after both right and left colectomies while achieving a tension-free colorectal anastomosis slowing the transit and increasing the absorption time, resulting in better stool consistency and quality of life compared with an ileorectal anastomosis.

**Methods:**

This technique was performed in a 41-year-old woman with Goblet cell adenocarcinoma of the appendix with peritoneal metastasis. The transverse colon is rotated anticlockwise over the axis of the middle colic vessels toward the left parietocolic flank and relocated to the usual position of the descending colon.

**Results:**

After 1 year of follow-up, the patient led a normal life without parenteral nutrition with five bowel movements per day and a weight gain of 15%.

**Conclusions:**

The use of an antiperistaltic transverse coloplasty may be worthwhile to perform in cases of extensive bowel resections during cytoreductive surgery leading to short-bowel syndrome to avoid a permanent stoma or intestinal failure and improve patient outcomes.

**Supplementary Information:**

The online version contains supplementary material available at 10.1245/s10434-023-14165-0.

Due to the loss of colonic reabsorption and/or rectal reservoir function, extensive colorectal resections may have poor outcomes, especially when accompanied by short-bowel syndrome. Although reabsorption has been partially preserved in some indications by preserving the cecum and performing a cecorectal anastomosis, colonic and ileal pouches have been proposed to maintain some reservoir function. In cases of carcinomatosis, in addition to extensive colorectal resections, extensive small-bowel resections also are performed; frequently the cecum cannot be preserved. In these cases, antiperistaltic transverse coloplasty can be interposed between the ileum and the rectal stump. Although challenging, this reconstruction can increase reabsorption and improve quality of life.

As far as we know, this kind of reconstruction has not been the subject of any reports in the literature. We describe an antiperistaltic transverse coloplasty technique used during cytoreductive surgery and HIPEC to improve reabsorption and quality of life following extended small-bowel resection and right and left colectomies.

## Technique

We conducted this technique in a 41-year-old female after performing resection of small bowel with a remaining small bowel of 1.8 meters, both right and left colectomies, hysterectomy, double adnexectomy, rectal anterior resection and central lymphadenectomy due to Goblet cell adenocarcinoma of the appendix with peritoneal metastasis.

The creation of a tension-free anastomosis after both left and right colectomies can be challenging and requires expertise and anatomical knowledge.^1^ To avoid tension between the remaining bowel ends, many surgeons would elect to perform a total colectomy. However, this results in deterioration of intestinal function with frequent bowel movements that persist in the long term and have an adverse effect on quality of life.^2^

**Fig. 1:**
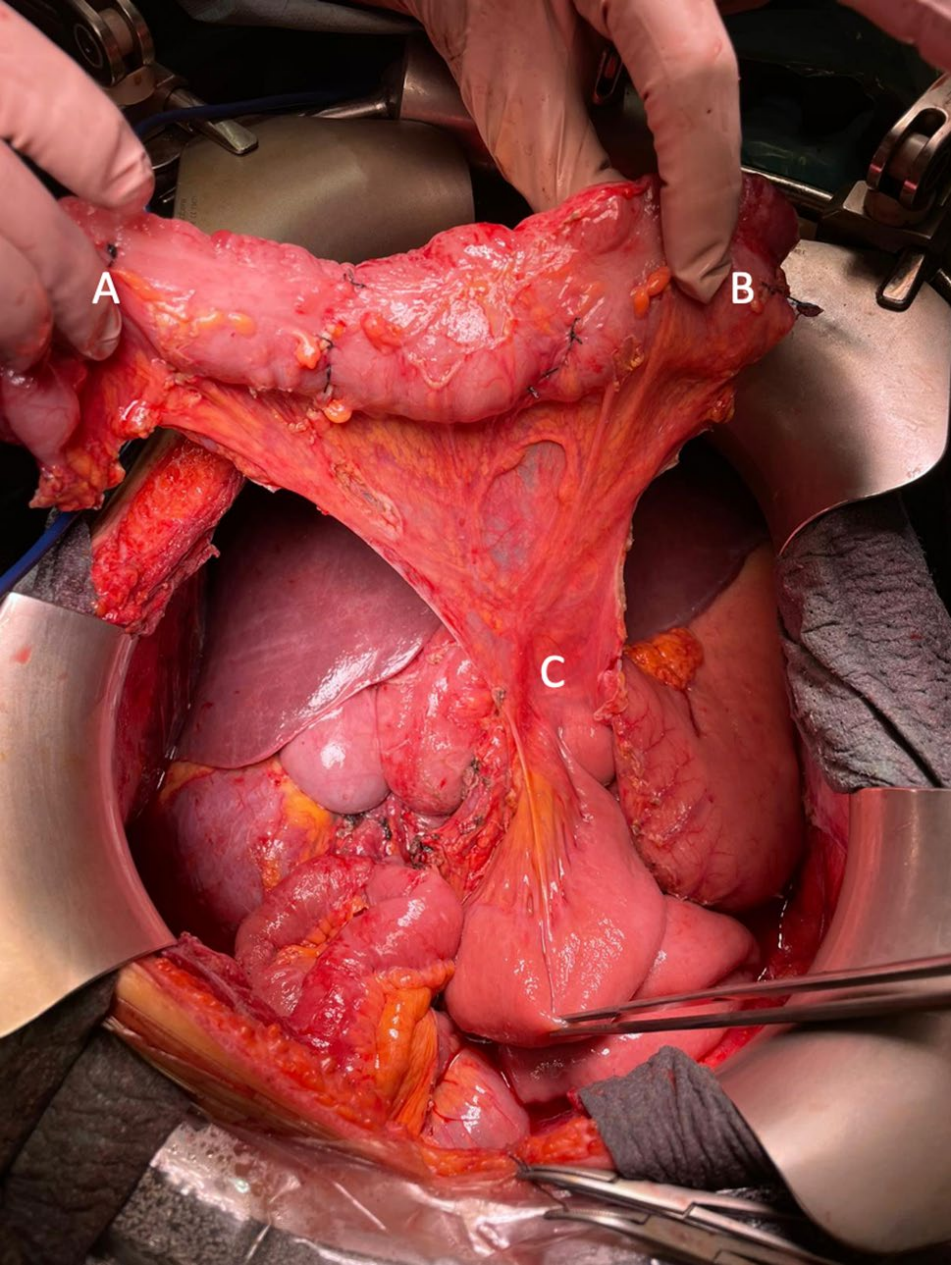
Exposure of the preserved transverse colon segment. (A) Hepatic flexure of the colon. (B) Splenic flexure of the colon. (C) Middle colic vessels preserved

**Fig. 2:**
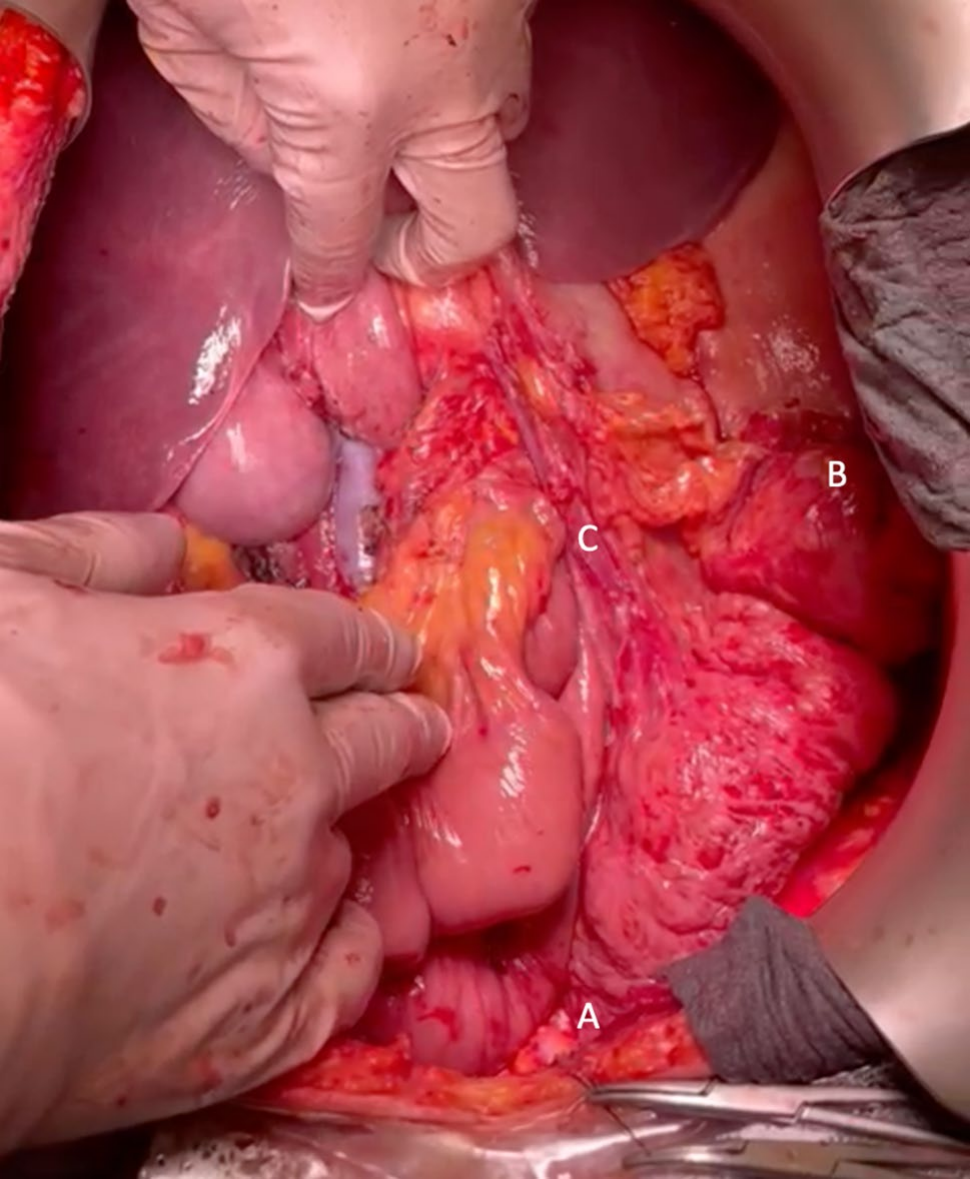
Anticlockwise rotation of the transverse colon. (A) Hepatic flexure of the colon. (B) Splenic flexure of the colon. (C) Middle colic vessels preserved

In our case, after the resection was concluded, intestinal transit was restored by performing an antiperistaltic transverse coloplasty before the HIPEC procedure. To perform this technique, middle colic vessels and their distal branches need to be preserved (Fig. 1). After assessing its correct vascularization, transverse colon is rotated anticlockwise over the axis of the middle colic vessels toward the left parietocolic flank. After that, the splenic flexure should remain in its position while the hepatic flexure reaches the middle rectum, and all of the transverse colon gets relocated to the usual position of the descending colon (Fig. 2). In this position, the posterior, mesocolic layer is transferred to an anterior position (Video 1). An anastomosis is then performed between the remnant small bowel and the splenic flexure of the colon. A colorectal anastomosis is constructed between the hepatic flexure of the colon and the rectum, with the colon in an antiperistaltic position.

## Results

The patient exhibited a favorable postoperative course during 1-year, follow-up period. Despite possessing a 1.8-meter remnant of small intestine without an ileocecal valve, implementation of a low-fiber diet, low-oxalate diet, and oral supplementation with fiber-free oligomeric hyperprotein hypercaloric formula (Survimed^®^) resulted in the patient attaining a fully normalized lifestyle with five daily bowel movements and a weight gain of 15%.

## Discussion

Short-bowel syndrome (SBS) causes structural changes in the intestine, which interfere with its main functions of digestion and absorption due to reduced surface area. The inadequate length of the small intestine increases the risk of developing intestinal insufficiency or failure in patients with SBS, which depends on the degree of malabsorption.^3^ After extensive intestinal resection, a compensatory adaptation of the residual intestine occurs physiologically, both at a structural and functional level, in order to enhance nutrient absorption and delay gastrointestinal transit.^4^

The length of the remaining small bowel is crucial for postsurgical outcomes, but the colon can be safely removed if the small bowel is intact. Therefore, the preservation of the colon is not a primary concern for surgeons.^5^ However, new studies indicate that, in the case of restoration of jejune-colonic anastomosis, the colon plays an important role in reducing the need for parenteral support, ultimately improving survival and quality of life. This highlights the significance of the colon alongside the small bowel in postsurgical outcomes.^1–3^

By performing this technique, at least 28% of the colon is salvaged according to Cummings classification and make it equivalent to a type 2 SBS.^6^ These patients also are referred to as SBS with a colon-in-continuity (SBS-CiC).^5^ Type 2 patients having the majority of the colon but no ileocecal valve can avoid parenteral nutrition with a minimum of 65 cm of remaining small bowel.^4,7^ With the preservation of colon-in-continuity, intravenous supplementation is unnecessary. SBS-CiC can offset malabsorption by increasing oral food intake or using pharmacotherapeutics, thereby avoiding intestinal failure.^5^

Furthermore, patients with extensive intestinal resections are at risk of bacterial overgrowth of the small intestine, worse absorption of water and sodium, and malabsorption due to fast transit and reduced absorption surface area.^5^ This results in a lower quality of life and worse stool consistency.^2^

Good intestinal function may also be preserved by salvaging any large intestine segments when extensive colon resection is added. Because the proximal segment also may serve as a reservoir in place of an ileal pouch, the shorter the rectal remnant, the more prudent the preservation, it stands to reason.^8^

Antiperistaltic transverse coloplasty allows for preservation of the transverse colon while achieving a tension-free colorectal anastomosis. The antiperistaltic placement of the colon slows the transit while increasing the absorption time, which might result in a retarded transit, increased intestinal absorption, better consistency of the stool, and quality of life compared with an ileorectal or jejunorectal anastomosis while avoiding intestinal failure.^6,9^

This technique enables us to concentrate on improving the quality of the remaining life in young patients with poor life expectancy after extensive cytoreductive surgery rather than having to perform a permanent ileostomy.^8^

## Conclusions

To avoid a permanent stoma or intestinal failure after cytoreductive surgery, an antiperistaltic transverse coloplasty may be worthwhile to perform whenever possible. An oncologic surgeon should be adept at this technique, because it might be the only workable solution for the saved segment.

### Supplementary Information

Below is the link to the electronic supplementary material.

Supplementary file1 (MP4 10692 KB)
